# Cooperative Quantum-Behaved Particle Swarm Optimization with Dynamic Varying Search Areas and Lévy Flight Disturbance

**DOI:** 10.1155/2014/370691

**Published:** 2014-03-03

**Authors:** Desheng Li

**Affiliations:** Anhui Science and Technology University, Fengyang, Anhui 233100, China

## Abstract

This paper proposes a novel variant of cooperative quantum-behaved particle swarm optimization (CQPSO) algorithm with two mechanisms to reduce the search space and avoid the stagnation, called CQPSO-DVSA-LFD. One mechanism is called Dynamic Varying Search Area (DVSA), which takes charge of limiting the ranges of particles' activity into a reduced area. On the other hand, in order to escape the local optima, Lévy flights are used to generate the stochastic disturbance in the movement of particles. To test the performance of CQPSO-DVSA-LFD, numerical experiments are conducted to compare the proposed algorithm with different variants of PSO. According to the experimental results, the proposed method performs better than other variants of PSO on both benchmark test functions and the combinatorial optimization issue, that is, the job-shop scheduling problem.

## 1. Introduction

PSO, originally introduced by Kennedy and Eberhart [1], has become one of the most important swarm intelligence-based algorithms. The unique information diffusion and interaction mechanism of PSO enable it to solve many problems with good performance at low computational cost. Hence, in the past decades, PSO is imported to solve the problems of numerical optimization [2], combinatorial optimization [3], controller optimization [4, 5], and software design optimization [6].

Among the applications, function optimization has been often chosen to check the performance of them, because benchmark functions are not only well described in literature such as their properties, locations, and values of the optimal solutions, but also have many different versions that can rove different capabilities of optimizer [7, 8]. However, according to the state of art in the relevant research [9] and in spite of its superior performance, PSO is even not a global optimization algorithm. To overcome this defect, some randomizing techniques are employed into the design of PSO, such as chaos [10] and quantum behavior [11, 12], to accelerate the global convergence of the algorithms. In literatures [11, 12], Sun et al. proposed a quantum-behaved PSO (QPSO) algorithm, which can be guaranteed theoretically to find optimal solution in search space. The experimental results on some widely used benchmark functions show that the QPSO works better than standard PSO and should be a promising algorithm.

Like all other intelligence algorithms [8], escaping from the local optimum and preventing premature convergences are two inevitable difficulties in implementation. Especially, as dimensionality increases, these kinds of problems become more complex and the possibility for finding global optimum sharply decreases. Nevertheless, some applications really need to probe the global optimal solutions rather than local ones, such as function optimization and clusters structure optimization. Hence, the main motivation of this research is to find a solution to make the PSO adapt to the multidimension and difficult problems, for example, NP hard ones.

This paper proposes a novel particle swarm algorithm with two different methods to reduce the search space. One is called Dynamic Varying Search Area (DVSA), which takes charge of limiting the ranges of particles' activity, and the other is cooperative strategy, which divides the candidate solution vector into small subswarms. Moreover, in order to escape the local optima, Lévy flights are used to generate the stochastic disturbance in the movement of particles.

The remainder of this article is organized as follows.  Section 2 provides a brief review on the related algorithms.  Section 3 proposes the CQPSO-DVSA-LFD and gives the main flow of the algorithm. Sections 4 and 5 illustrate the inner mechanisms, Dynamic Varying Search Area (DVSA), and Lévy Flights Disturbance (LFD), respectively.  Section 6 describes the experimental framework and then presents and discusses the numerical results from the trails. Finally, Section 7 offers our conclusions and future work.

## 2. Review on Related PSO Algorithms

### 2.1. PSO

PSO algorithm was first introduced by Kennedy and Eberhart [1] as a simulation of the flock's behavior but quickly evolved into one of the most powerful optimization algorithms in the computational intelligence field. The algorithm consists of a population of particles that are flown through an *n*-dimensional search space. The position of each particle represents a potential solution to the optimization problem and is used in determining the fitness (or performance) of a particle. In each generation of iteration, particle in swarm can be updated by the values of the best solution found by it and the one found by the whole swarm by far according to the following equation:

(1) vidt+1=ω×vidt+c1×r1×(Pidbest−Pidt) +c2×r2×(Pgdbest−Pidt),Pidt+1=Pidt+vidt+1,

where *v*_id_^*t*^ denotes the particle's velocity in *t* generation and *P*_id_^*t*^, *P*_id_^best^, and *P*_gd_^best^ are the particle's position, personal historical best position, and swarm's global best position in *t* generation, respectively.

### 2.2. CPSO

In practice, most variants of standard PSO suffer from the curse of dimensionality, which may directly reduce to the performance deterioration. Therefore, it also causes the failure of finding the global optimum of a highdimensional problem. One effective way is to decompose the large search space into smaller swarms in lower dimensional vector space, that is, to employ cooperative strategy. Based on the rationale of Cooperative Coevolutionary Genetic Algorithm (CCGA) in [13], van den Bergh and Engelbrecht introduced the Cooperative PSO that employs a kind of cooperative behavior to significantly improve the performance of the original algorithm [9]. Compared to basic single swarm PSO, both robustness and precision are improved and guaranteed. The key idea of CPSO is to divide all the *n*-dimension vectors into *k* subswarms. So the front *n/k *swarms are   ⌈*n*/*k*⌉-dimensional and the *k* − (*n*/*k*) swarms behind have ⌊*n*/*k*⌋-dimensional vectors. In each pass of iteration, the solution is updated based on *k* subswarms rather than the original one. When the particles in one subswarm complete a search along some component, their latest best position will be combined with other subswarms to generate a whole solution.

The function *b* shown in (2) performs exactly like this: it takes the best particle from each of the other subswarms and concatenates them, splicing in the current particle from the current subswarm *j* in the appropriate position. According to this function, the composition of *P*_id_^best^, *P*_gd_^best^, and *P*_cgd_^best^ can be calculated based on (3)–(5). Due to the employment of this component, the particles in each subswarm therefore update their global best position by (6)-(7), which is the result associated with minimal fitness value of their local best positions and global best positions of electoral swarm:

(2) b(u,Z)=(S1·Pgdbest,…,Su−1·Pgdbest,Z,Su·Pgdbest,…,Sk·Pgdbest), 1≤u≤k,

(3) b(u,Su·Pidbest) =argmin  fitness(b(u,Su·Pidbest),b(u,Su·Pidt)),1≤id≤s, 1≤u≤k,

(4) b(u,Su·Pgdbest)=argmin  fitness(b(u,Su·Pidt)),1≤id≤s, 1≤u≤k,

(5) b(u,Su·Pcgdbest) =argmin  fitness(b(u,Su·Pidbest),b(u,Su·Pcgdbest)),1≤id≤s, 1≤u≤k,

(6) vidt+1=ω×vidt+c1×r1×(Pidbest−Pidt)+c2×r2 ×(Pgdbest−Pidt)+c2×r2×(Pcgdbest−Pidt),

(7) $$\begin{array}{c} P_{\text{id}}^{t+1}=P_{\text{id}}^{t}+v_{\text{id}}^{t+1}. \end{array}$$

### 2.3. QPSO

In literature [14], Liu et al. proposed a Quantum Particle Swarm Optimization (QPSO), which discards the velocity vector of original PSO and consequently changes the updating strategy of particles' position to make the search more simple and efficient. QPSO is the integration of quantum computing and PSO. The QPSO is based on the representation of the quantum state vector. It applies the probabilistic amplitude representation of quantum to the coding of particles, which makes one particle represent the superposition of many states and uses quantum rotation gates to realize the update operation.

**Figure 1:**
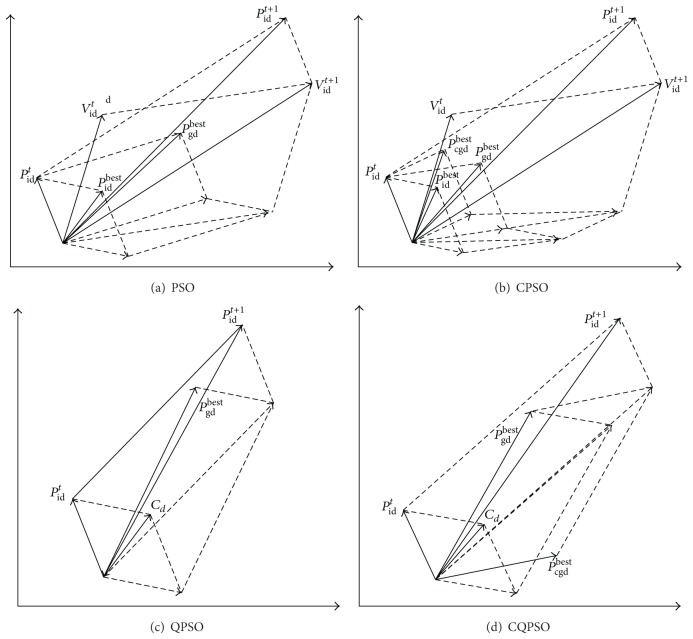
Particle movement principle of PSO, CPSO, QPSO, and CQPSO.

The iterative equation of QPSO is very different from that of PSO. Besides, unlike PSO, QPSO needs no velocity vectors for particles and also has fewer parameters to adjust, making it easier to implement. This can be seen clearly in Figure 1.

The updated strategy of particles' position of QPSO is as follows:

(8) $$\begin{array}{c} P_{\text{id}}^{t+1}=\varphi \times P_{\text{id}}^{\text{best}}+\left(1-\varphi \right)\times P_{\text{gd}}^{\text{best}}\pm \beta \times \left|C_{d}-P_{\text{id}}^{t}\right|\times \ln\left(\frac{1}{u}\right), \end{array}$$

where *N* is population of particles; *D* is dimension of problem; *P*_id_^*t*^ is position of particle; *P*_id_^best^ is local best position; *P*_gd_^best^ is global best position; *C*_*d*_ = (1/*N*)∑_*i*=1_^*N*^ *P*_id_, *d* = 1,…, *D*, *C* = *C*_1_,…;*C*_*d*_ is mean local best positions.

### 2.4. CQPSO

In CQPSO algorithm proposed in [15], an electoral swarm is generated by the voting of primitive subswarms and also participates in evolution of swarm, whose candidate particles come from primitive subswarms with variable votes. In reverse, the number of selected particles could also impact the voting of the primitive subswarms, such as the total number of candidates and quota of selected ones. The selected candidates could share their components with best segments of position, which are then composed into a new particle position to participate in the combining of positions. Like the treatment in our previous work, a new component of particle's position is also imported, that is, *P*_cgd_^best^, denoting the electoral best position composed by the dimensions of elected candidates.

The updating strategy of particles' position of CQPSO is as follows:

(9) Pidt+1=φ×Pidbest+ψ×Pgdbest+(1−φ−ψ)×Pcgdbest±β ×|Cd−Pidt|×ln⁡⁡(1u).

## 3. Proposed Algorithm: CQPSO-DVSA-LFD

In this paper a novel particle swarm algorithm with two different methods is proposed to reduce the search space. One is called Dynamic Varying Search Area (DVSA), which takes charge of limiting the ranges of particles' activity and the other is cooperative strategy, which divides the candidate solution vector into small subswarms. The illustration of DVSA will be introduced in Section 4.

On the other hand, theoretically, CQPSO algorithm could solve any problem by QPSO. However, due to the possibility of trapping in a case that all subswarm could not find the optimum, CQPSO could also reach the current minimum. To avoid this kind of stagnation, we employ a stochastic disturbance method, that is, Lévy flights disturbance, to generate a random movement of the stagnant subswarms. The details of Lévy flights disturbance will be introduced in Section 5.

Differently with other similar methods, we use the output parameters of Lévy flights to intervene the position change directly, which can be seen in (10) as follow, where ${\text{Angle}}_{\text{L}\overset{´}{\text{e}}\text{vy}}$ and ${\text{Step}}_{\text{L}\overset{´}{\text{e}}\text{vy}}$ are the output parameters of Lévy flights which are randomly generated, while *ε*_1_, *ε*_2_, and *ε*_3_ are the parametric empirical coefficient:

(10) Pidt+1=φ×Pidbest+ψ×(Pgdbest+ε1×AngleLévy) +(1−φ−ψ)×(Pcgdbest+ε2×AngleLévy) ±β×|(Cd+ε3×StepLévy)−Pidt|×ln⁡⁡(1u).

Based on the above introduction, we can now present the proposed CQPSO-DVSA-LFD algorithm in the following steps in Algorithm 1.

## 4. Dynamic Varying Search Area (DVSA)

### 4.1. Rationale of Dynamic Varying Search Area (DVSA)

As we know, complexity of optimization problem does not only rely heavily on the objective/constraint function but also relates to its search area. Simply speaking, subjected to the same objective/constraint function, the larger the search area is, the harder it can find the solution [16]. Based on this idea, to change the search area dynamically, or say it reduces, is necessary to accelerate the processing of algorithm. On the other hand, when the search area reduced, the populations of subswarms are unnecessary as big as previous ones.

Given an optimization function:

(11) $$\begin{array}{c} \min\;f\left(x\right),\;x={\left(x_{1},x_{2},\ldots ,x_{N_{d}}\right)}^{T}\in S\subseteq R^{N_{d}}, \end{array}$$

where *S* = [*a*_1_, *b*_1_] × [*a*_2_, *b*_2_] × ⋯×[*a*_*N*_*d*__, *b*_*N*_*d*__], the basic rationale of Dynamic Varying Search Area (DVSA) could be illustrated as the following description. Firstly, assume that *N*_*p*_ cooperative subswarms probe is in the search space. When the minimal distances between optimal individuals of each subswarm reached a threshold, according to the maximum likelihood estimation, the hypothesis that the real optimal solution is in the area surrounded by these particles was established. Then reduce the previous search area *S* to *S*′, generate a new swarm with same subswarms on *S*′, and decrease the populations meanwhile. Finally, repeat the above procedures until satisfying the end condition.

**Figure 2:**
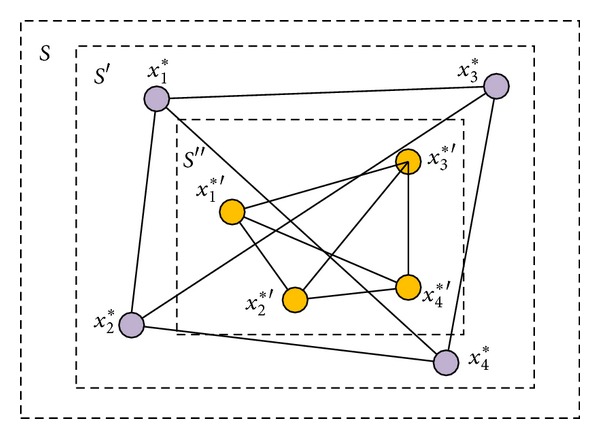
A case of DVSA.

Consider the vector *x* before the *r*th reduce, where the *i*th component *x*_*i*_ ranges over [*a*_*i*_^*r*−1^, *b*_*i*_^*r*−1^]. Then *x* could be expressed as *x* ^*r*−1^ ∈ [*a*_*i*_^*r*−1^, *b*_*i*_^*r*−1^].  Figure 2 exemplifies the case that four cooperative subswarms reduce their search area. First, they probe the solution in *S* and get the best particles *x*_1_*, *x*_2_*, *x*_3_*, and *x*_4_* included in *S*′. So the search area becomes *S*′. The next time of reduction to *S*′′ is the same procedure.

### 4.2. Condition of DVSA

In this part, we will give the condition when the DVSA occurs. Suppose there exist *N*_*p*_ subswarms, the best particles set found is written as

(12) xbr−1={xbr−1,1,xbr−1,2,…,xbr−1,Np},xbr−1,p=(xb1r−1,p,xb2r−1,p,…,xbNdr−1,p),p=1,2,…,Np.

Now, let us consider the distance among them:

(13) $$\begin{array}{c} D\left(x_{b}^{r-1,i},x_{b}^{r-1,j}\right)={||x_{b}^{r-1,i},x_{b}^{r-1,j}||}_{2},\\ D_{}^{r-1}=\underset{x_{b}^{r-1,i},x_{b}^{r-1,j}\in x_{b}^{r-1}}{\max}D\left(x_{b}^{r-1,i},x_{b}^{r-1,j}\right), \end{array}$$

where ||·||_2_ is the 2-norm on corresponding search area.

When *D* ^*r*−1^ reached a small threshold, according to the maximum likelihood estimation, the hypothesis that the real optimal solution is in the area surrounded by these particles was established. So the latter search can be performed around these particles.

In light of this, we can give the condition of DVSA as shown below

(14) $$\begin{array}{c} D^{r-1}<\lambda \cdot {||a_{}^{r-1}-b_{}^{r-1}||}_{2},\;\lambda \in \left(0,1/N_{p}\right]. \end{array}$$

In other words, if the above equation is satisfied, then change the search area of the next generation of subswarms until the DVSA occurs again. *λ* can be a fixed number, but more often, it is a parameter that can be changed adaptively according to the results of evolution.

Let us consider the search area after reduction. Note that after the *r*th reduce, *x*_*i*_ ranges over [*a*_*i*_^*r*^, *b*_*i*_^*r*^]. Then the upper/lower bounds are defined by the following equation:

(15) air=min⁡{xbir−1,p}−ξ·(bir−1−air−1),bir=min⁡{xbir−1,p}+ξ·(bir−1−air−1), ξ∈(0,1].

To guarantee that the new search area is not larger than the previous area, the above equation should be modified as follows:

(16) $$\begin{array}{c} a_{i}^{r}=\{\begin{array}{cc} a_{i}^{r-1}, & a_{i}^{r}<a_{i}^{r-1},\\ a_{i}^{r}, & \text{otherwise}, \end{array}\\ b_{i}^{r}=\{\begin{array}{cc} b_{i}^{r-1}, & b_{i}^{r}<b_{i}^{r-1},\\ b_{i}^{r}, & \text{otherwise}. \end{array} \end{array}$$

### 4.3. Policy of Population Scale Adjustment

The computational complexity also relies heavily on the scale of the population of the swarm/subswarm. In general, the more take time about particle evaluation is, the more computation takes place. Hence, under the permission of optimization performance, it is necessary to cut down the population of subswarms.

In this article, we will follow a traditional method called search granularity. Take the particle after the *r*th reduce for instance, whose *i*th component *x*_*i*_ ranges over [*a*_*i*_^*r*^, *b*_*i*_^*r*^]. The distance of this interval can be written as (17) which reflects the refined effort of search. If the distance among the solutions is small, we can say that search granularity is small and vice versa. From the real experience, the bigger the swarm is, the less distance exists among the particles, which also lessen the search granularity:

(17) $$\begin{array}{c} d_{i}^{r}=b_{i}^{r}-a_{i}^{r}. \end{array}$$

Furthermore, if it is asked that the search granularity on [*a*_*i*_^*r*^, *b*_*i*_^*r*^] should be 1/*N*_*ik*_, the population scale of subswarm can be determined by the below equation:

(18) $$\begin{array}{c} N_{i}^{r}=\left\lfloor \overset{N_{d}}{\underset{k=1}{\prod }}N_{ik}\cdot d_{i}^{r}\right\rfloor , \end{array}$$

where ⌊·⌋ is the floor function. When the search area decreases, the population of the related subswarm also becomes small.

### 4.4. Theoretical Analysis

In this subsection, an analysis of the convergence of CQPSO-DVSA-LFD is provided. We discuss it from two perspectives, that is, search area and population of swarms.

Firstly, we analyze the variance of interval measure caused by two neighboring reduces. According to the policy of DVSA, it can be described as follows according to (19), (20):

(19) $$\begin{array}{c} b_{i}^{r}-a_{i}^{r}\leq b_{i}^{r-1}-a_{i}^{r-1}. \end{array}$$

Without loss of generality, let

(20) $$\begin{array}{c} b_{i}^{r}-a_{i}^{r}=k_{i}^{r}\cdot \left(b_{i}^{r-1}-a_{i}^{r-1}\right),\;k_{i}^{r}\in \left(0,1\right], \end{array}$$

then

(21) bir−air=kir·(bir−1−air−1)=kir·kir−1(bir−2−air−2)=⋯=Kir·(bi−ai),Kir=∏j=1rkir≤min⁡{ki1,ki2,…,kir}.

From formula (21), we can see that when search area varies, the reduced area becomes the *k*_*i*_^*r*^ times of origin area. So when several generations of this procedure happen, the final area could be heavily reduced with the considerable promotion of efficiency.

Secondly, in consideration of swarm populations, we can get the result from

(22) Njr=⌊∏i=1NdNji·dir⌋=⌊∏j=1NdNji·(bir−air)⌋=⌊∏j=1NdNji·Kir(bir−air)⌋<⌊∏j=1NdNji·(bi−ai)⌋.

The above inference shows that as the search area decreases, the related populations of swarms can also be cut down with a certain rate.

## 5. Lévy Flights Disturbance

The technique of random disturbance is often imported to improve the performance of PSO or QPSO. When QPSO was proposed, the Gaussian and Cauchy probability distribution disturbances have been used to avoid premature convergence. In [17], the random sequences in QPSO were generated using the absolute value of the Gaussian probability distribution with zero mean and unit variance. Based on the characteristic of QPSO, the variables of the global best and mean best positions are mutated with Cauchy distribution, and an adaptive QPSO version was proposed in [14].

In this paper, another random method, Lévy flights, is employed to do this work. Lévy flights, named after the French mathematician Paul Pierre Lévy, are Markov processes. After a large number of steps, the distance from the origin of the random walk tends to a stable distribution. Lévy flights, which can be characterized by an inverse square distribution of step length, may optimize the random search process when targets are scarce and at scarcity of resources. In contrast, Brownian motion is usually suitable for the case when there is a need to locate abundant prey or targets. These traits of two random walks inspired us to improve our swarm intelligence optimization, where Lévy flights can improve the ability of “exploration” while Brownian motion benefits the “exploitation.”

Mathematically, Lévy flights are a kind of random walk whose step lengths meet a heavy-tailed Lévy alpha-stable distribution, often in terms of a power-law formula, *L*(*s*)~|*s*|^−1−*β*^, where 0 < *β* ≤ 2 is an index. A typical version of Lévy distribution can be defined as [18]

(23) L(s,γ,μ)={γ2πexp⁡[−γ2(s−μ)]1(s−μ)3/2,0<μ<s<∞;0,s≤0.

At the change of **β**, this can evolve into one of Lévy distributions, normal distributions; and Cauchy distributions.

**Figure 3:**
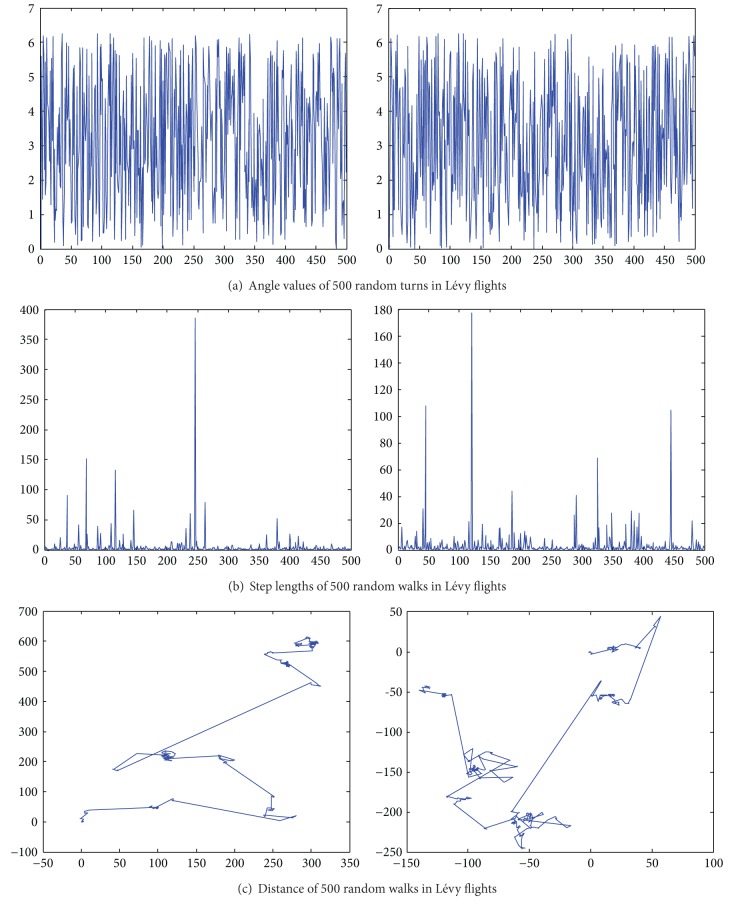
2D Lévy flights in 500 steps.

Taking the 2D-Lévy flights for instance, the steps follow a Lévy distribution as in Figure 3(b), while the directions of its movements meet a uniform distribution as in Figure 3(a). As shown in Figure 3(c), an instance of the trajectory of 500 steps of random walks is obeying a Lévy distribution. Note that the Lévy flights are often more efficient in exploring unknown and large-scale search space than Brownian walks. One reason for this argument is that the variance of Lévy flights *δ* ^2^(*t*) ~ *t* ^3−*β*^, 1 ≤ *β* ≤ 2 increases faster than that of Brownian random walks, that is, *δ* ^2^(*t*) ~ *t*. Also, compared to Gaussian distribution, Lévy distribution is advantageous since the probability of returning to a previously visited site is smaller than that for a Gaussian distribution, irrespective of the value of *μ* chosen.

From the update strategy of CQPSO-DVSA-LFD, we can draw a conclusion that all particles in CQPSO-DVSA-LFD will converge to a common point, leaving the diversity of the population extremely low and particles stagnated without further search before the iterations are over. To overcome the problem, we exert a disturbance generated by Lévy flights on the mean best position, global best position, and electoral best position when the swarm is evolving as shown in the following equation (24). To the local attractor, the hop steps in Lévy flights promise the random traversal in the search space. However, to the global and electoral best locations, they only need a slightly disturbance; that is, the angles meet a uniform distribution, to exploit the particles nearby

(24) $$\begin{array}{c} {C_{d}^{\prime }=C}_{d}+\varepsilon_{3}\times {\text{Step}}_{\text{L}\overset{´}{\text{e}}\text{vy}},\\ {P_{\text{gd}}^{{\text{best}}^{\prime }}=P}_{\text{gd}}^{\text{best}}+\varepsilon_{1}\times {\text{Angle}}_{\text{L}\overset{´}{\text{e}}\text{vy}},\\ {P_{\text{cgd}}^{{\text{best}}^{\prime }}=P}_{\text{cgd}}^{\text{best}}+\varepsilon_{2}\times {\text{Angle}}_{\text{L}\overset{´}{\text{e}}\text{vy}}, \end{array}$$

where *ε*_1_, *ε*_2_, and *ε*_3_ are a prespecified parameter, ${\text{Step}}_{\text{L}\overset{´}{\text{e}}\text{vy}}$ is a number in a sequence by Lévy flights, angle is the angles of directions in Lévy flights.

## 6. Experimental Studies

### 6.1. Experiments on Continuous Optimization Benchmarks

To study the search behavior and its performance of CQPSO-DVSA-LFD with other versions of PSO, such as plain PSO, CPSO, and CQPSO, some typical benchmark functions of continuous optimization are selected as the examples [19, 20].

**Figure 4:**
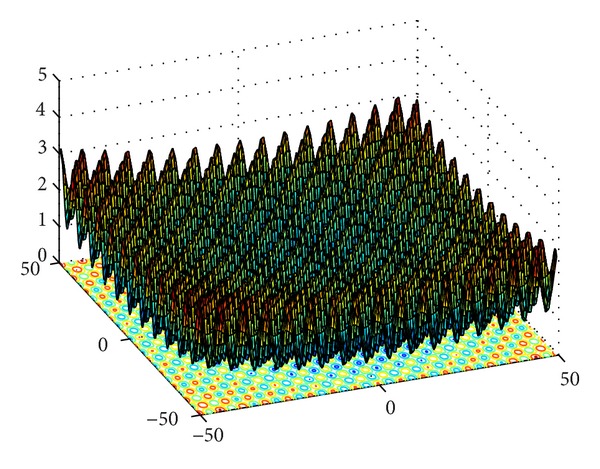
Surface landscape of Griewank function.

Rastrigin's function is frequently used as a test function to test the performance of optimization algorithms. Based on Sphere function, it uses cosine function to generate lots of local optimal points. It is a complex multimodal function, and optimization falls into the local optimum easily. Griewank function is a spin, inseparable, variable-dimension, multimode function as shown in Figure 4. At the increase of its dimension, the scope of local optimum gets narrower so that searching global optimum becomes easy relatively. Therefore, for Griewank function, it is harder to get solution in low dimension than in high dimension. Michalewicz function is a multimodal function with parameter *m* which changes the steepness of valleys. The Lévy number 8 function has one global minimum and, approximately, 125 local minima.

Computational results of variants of PSO used in the paper are qualitatively ranked in Table 1. From it, we can clearly get that the proposed CQPSO-DVSA-LFD algorithm performed greatly better than the plain PSO and QPSO. Also, compared to the basic Cooperative PSO (CPSO), the convergence property has been enhanced by the proposed techniques in the paper.

**Figure 5:**
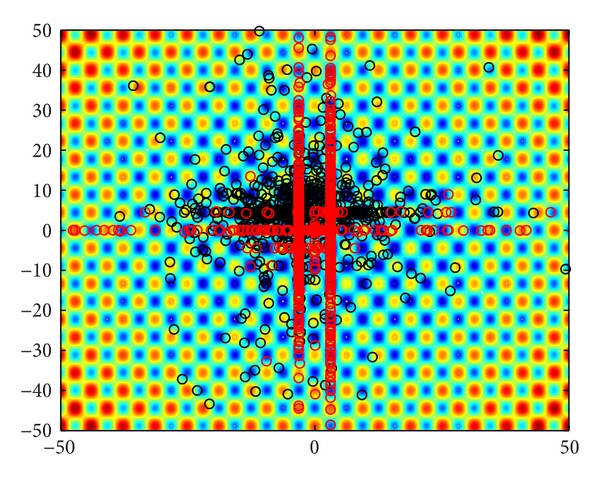
Particles in QPSO and CQPSO-DVSA-LFD.

In Figure 5, the black cycles denote the distribution of particles of 2-d Griewank function in QPSO under DVSA and LFD, while the red ones express that of CQPSO-DVSA-LFD with only two cooperative subswarms. It can be clearly seen that in CQPSO-DVSA-LFD, the search area in each generation of iteration is reduced dynamically into the potential rectangles along two red lines on horizontal/vertical directions. In addition, we can also find that the populations of the latter generations has been reduced obviously, which means the lower computational complexity meanwhile.

Moreover, the convergence ability is also investigated in our experiment.  Figure 3 illustrates the typical convergence of PSO, CPSO, QPSO, CQPSO, and CQPSO-DVSA-LFD on the benchmark Michalewicz function. From the figure, it can be seen that the varying curves of objective values using the CQPSO-DVSA-LFD descend much faster than that when using plain PSO and QPSO. In addition, the fitness values descent to lower level by using CQPSO-DVSA-LFD than CPSO due to the different mechanisms of simulated annealing and DVSA.

**Figure 6:**
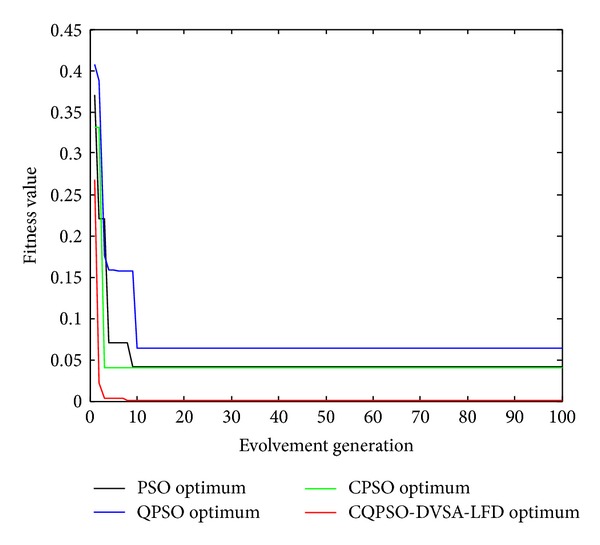
Evolution curves of Griewank function.

From Figure 6, the results of the experiments indicated that the proposed CQPSO-DVSA-LFD can lead to more efficiency and stability than plain PSO, QPSO, CPSO, and CQPSO.

### 6.2. Experiments on Combinatorial Optimization Problem

For the combinatorial optimization problem, we choose job-shop scheduling problem (JSSP) to test the performance of our algorithm. Job-shop scheduling problem (JSSP) is well known that it is in the family of NP-hard and NP-complete and has proven to be an impossible task for human schedulers. In the job-shop scheduling problem, finite jobs are to be processed by fix-numbered machines. Each job consists of a predetermined sequence of task operations, each of which needs to be processed without preemption for a given period of time on a given machine. But, tasks of the same job could not be processed concurrently and each job must be on each machine exactly once. Moreover, each operation cannot be commenced until the processing is completed, if the precedent operation is still being processed. A schedule is an assignment of operations to time slots on a machine. The makespan is the maximum completion time of the jobs and the objective of the JSSP is to find a schedule that minimizes the makespan.

**Figure 7:**
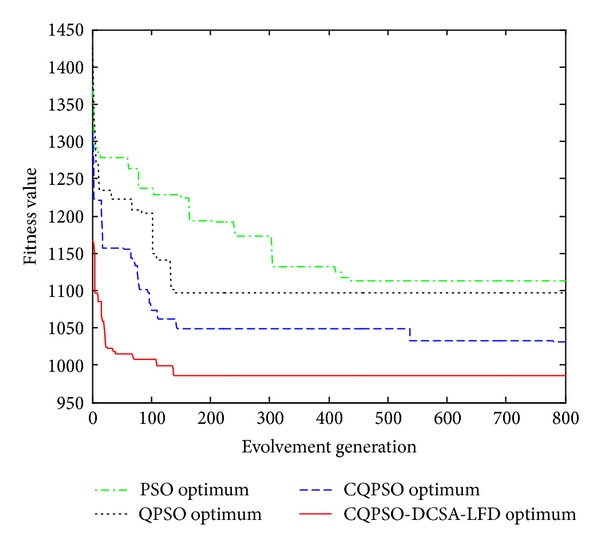
Evolution curves of JSSP.

**Figure 8:**
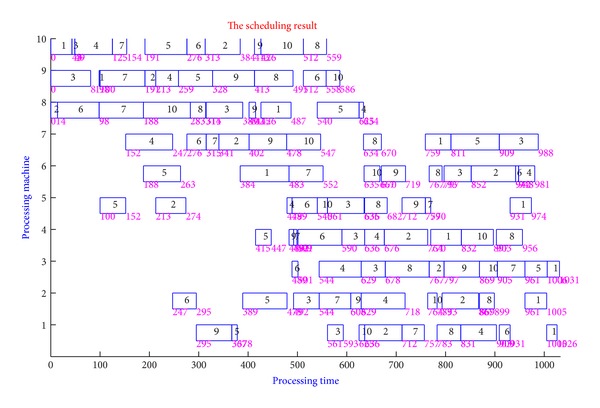
GANT graph of 6∗6 JSSP generated by CQPSO-DVSA-LFD.

The experiments on JSSP are performed on 6∗6, 10∗10, and 20∗5 instances, respectively, and its convergence efficiency and GANT are shown in Figures 7 and 8. We can see that the convergence rate of CQPSO-DVSA-LFD is clearly faster than other PSO algorithms from our simulation solution.

## 7. Conclusions and Future Work

In this paper, we proposed a CQPSO-DVSA-LFD algorithm which is a combination of QPSO, cooperative mechanisms which are used to find better particles in shorter time, and two ways to reduce the search space. One is called Dynamic Varying Search Area (DVSA), which takes charge of limiting the ranges of particles' activity; the other is cooperative strategy, which divides the candidate solution vector into small subswarms. Moreover, to help escape from local optima, a disturbance generated by Lévy flights is embedded as a hybrid strategy. Computational results and comparisons on both continuous optimization benchmarks and JSSP problem show that it outperforms other related algorithms.

Future research may include a further investigation of the algorithm to solve other problems. It is also worthwhile to tune Lévy flights disturbance approaches and analyse the performance about the improvement by this random disturbance. The parameter setting optimally and adjustment of algorithm termination conditions are also one of our focuses.

## Figures and Tables

**Algorithm 1:**
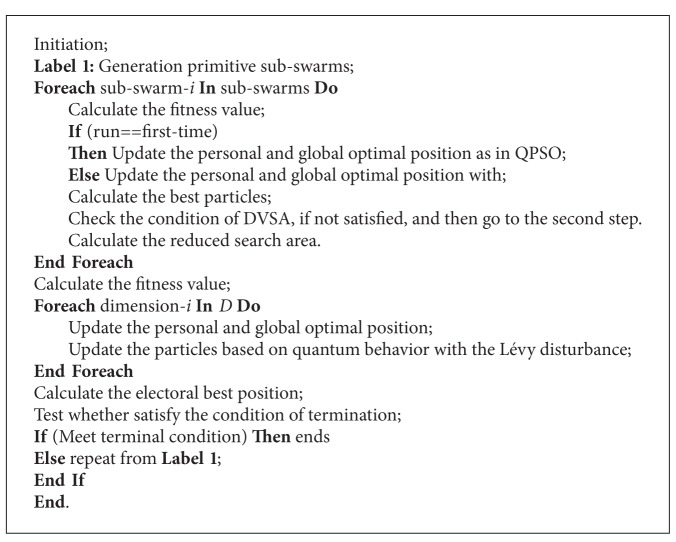
Pseudocode of CQPSO-DVSA-LFD.

**Table 1:** Results of functions optimization in benchmark.

Functions	PSO			QPSO			CQPSO			CQPSO-DVSA-LFD		
	Minimum	Maximum	Average	Minimum	Maximum	Average	Minimum	Maximum	Average	Minimum	Maximum	Average
Rastrigin's	6.12*E* − 06	9.00*E* + 00	4.67*E* + 00	3.01*E* + 00	8.90*E* + 01	4.91*E* + 01	**2.50*E* + 01**	1.25*E* + 02	7.52*E* + 01	**2.50*E* + 01**	**2.50*E* + 01**	**2.50*E* + 01**
Griewank	9.87*E* − 03	1.50*E* − 01	5.04*E* − 02	9.12*E* − 06	8.68*E* − 02	3.86*E* − 04	2.58*E* − 06	4.95*E* − 02	1.56*E* − 02	**0**	8.04*E* − 10	6.83*E* − 11
Michalewicz	−9.284715	−7.846484	−8.387755	−9.375576	−8.195449	−9.006959	−9.613477	−8.394369	−9.237160	**−9.660151**	**−9.660151**	**−9.660151**
Lévy	0.6663	4.6048	2.3496	0.1019	11.5187	2.2827	3.27*E* − 05	2.94*E* − 04	1.48*E* − 04	**2.01*E* − 05**	6.37*E* − 04	2.38*E* − 04

The bold values denote the stable approximate optimal solution compared to other algorithms.
